# Dual-Period Polarization-Dependent Diffraction Gratings Based on a Polymer-Stabilized Liquid Crystal

**DOI:** 10.3390/ma16237313

**Published:** 2023-11-24

**Authors:** Marta Kajkowska, Miłosz Sławomir Chychłowski, Sławomir Ertman, Piotr Lesiak

**Affiliations:** Faculty of Physics, Warsaw University of Technology, Koszykowa 75, 00-662 Warsaw, Poland; milosz.chychlowski@pw.edu.pl (M.S.C.); slawomir.ertman@pw.edu.pl (S.E.)

**Keywords:** liquid crystal, polymer, polymer-stabilized liquid crystal, dual-period diffraction grating, polarization

## Abstract

In this paper, we demonstrate the first ever dual-period diffraction gratings that do not require electrical tuning to obtain the effect of period change. Our method allows for multiplication of the base period by proper modification of the subsequent slits of the grating. The proposed elements are fabricated by selective photopolymerization of a composite based on a nematic liquid crystal. The gratings are formed by polymer stabilization of a liquid crystal in different orientations of the molecules in selected grating slits to allow for period manipulation. The operating principle is based on changing the phase delay introduced by the slits depending on polarization direction of incident light with respect to the director in each type of slit, which allows to change the grating’s period. The proposed technique was successfully utilized to obtain diffraction gratings with either doubling or tripling of the period.

## 1. Introduction

Composites consisting of liquid crystals (LCs) and polymers have been widely studied over the years. Such materials are usually categorized as polymer-stabilized LCs (PSLCs) or polymer-dispersed LCs (PDLCs), depending on the LC to monomer ratio. In case of PSLCs, the polymer network is formed in between LC molecules and stabilizes the molecular arrangement, as the concentration of monomer is quite low (<10%) [1,2]. Such a network allows to not only expand the temperature stability of the LC phase [3,4,5,6], but to also preserve the desired orientation of LC molecules that was initially obtained under the influence of an external factor such as an electric field [7,8,9,10,11,12]. When it comes to PDLCs, the polymer concentrations typically range from ~20% to 80% [1]. In such composites, the LC and polymer separate during the polymerization process, and thus LC droplets dispersed in the polymer matrix are formed [13]. In contrast to PSLCs, the molecules within LC droplets are oriented randomly, and thus the material is highly scattering when no electric field is applied.

Polymer-doped LCs have been demonstrated to be suitable for applications in optics and photonics, especially when it comes to tunable lenses [14,15,16], diffractive optical elements [3,12,17,18,19,20,21,22], LCDs [5,23,24,25], waveguides [8], and phase shifters [26,27,28]. Stabilization of LC’s molecular arrangement with a polymer network has been used to fabricate various types of lenses including Fresnel zone plates [17], and lenses with electrically tunable focal length [15,16]. However, a huge issue with such lenses is the fact that their performance is strongly dependent on the polarization of incident light. When it comes to diffraction gratings, it was demonstrated that such elements can be fabricated based on PDLCs irradiated with a periodic pattern, although external electric field was required for the gratings to operate properly, as otherwise the LC within the droplets was oriented randomly [29]. Other groups reported a method of forming PDLC-based [30] and PSLC-based [31] diffraction gratings by introducing the material into LC cells with periodic in-plane-switching electrodes. Additionally, LC-based diffraction gratings with no polymer added to the material have been demonstrated. In those cases, the gratings were either created by irradiation of photosensitive alignment layers [32,33,34,35,36,37,38] or by introducing the LC into LC cells with periodic electrodes [39]. Moreover, the diffraction efficiencies of the presented LC-based diffraction gratings were dependent on polarization of incident light and the value of applied voltage in cases of the ones with periodic electrodes. The concept of dual-period diffraction gratings has also been investigated [40], as such gratings can be highly beneficial for beam steering. Such gratings based on polymer-stabilized blue phase LCs have been demonstrated; however, the polymer was used only to provide stability of the material, and the gratings themselves were formed by periodic electrodes [21,22].

The main goal of the presented research was to provide a proof of concept for polymer-stabilized dual-period diffraction gratings operating for linearly polarized light. To achieve this, strong dependence of LC-based diffractive optical elements on polarization direction of incident light was utilized. The fabrication method was relatively simple, as it was based on selective polymer stabilization of the electrically controlled orientation of LC molecules in selected slits of each diffraction grating. As a result, stable structures with periodically variable molecular arrangements were formed and, unlike in the case of diffraction gratings based on PDLCs [29] or LC cells with periodic electrodes [21,22], no external electric field was required for them to operate. Additionally, the polymerization-induced increase in the thermal stability of the LC [6] makes the proposed fabrication technique superior to the one based on photoalignment. The fabricated samples were examined in a free space optical setup to prove the possibility of changing the gratings’ period by rotating the polarization direction of incident linearly polarized light. When it comes to application prospects, it is thought that a combination of polymer-stabilized LC-based polarization-dependent dual-period diffraction gratings, designed to exhibit different period changes, can be used as a main discriminator for a polarimeter [41,42,43,44,45]. Moreover, the proposed type of diffraction gratings can be utilized as a passive element in a spectrometer that allows to change the spectral resolution just by controlling the polarization of incoming light.

## 2. Materials and Methods

### 2.1. Material Preparation

The composite used in the research was based on a nematic LC 5CB (4-Cyano4′-pentylbiphenyl, >99.5%) doped with reactive monomer Bisphenol A dimethacrylate (Sigma Aldrich, St. Louis, MO, USA, >98%, CAS: 3253-39-2) and photoinitiator 4,4′-Bis(diethylamino)benzophenone (Sigma Aldrich, St. Louis, MO, USA, ≥99%, CAS: 90-93-7). The concentration of the dopant was 2.5 wt%, and the ratio between the monomer and photoinitiator was 9:1. The material was created by mixing the dopant compounds in desired proportions and then adding the LC to ensure a precise measurement of the dopants’ amount. Next, the composite was mixed in ultrasonic bath in temperature above the clearing point of 5CB for 30 min to provide uniform distribution of the compounds. The mixed material was introduced into 12 µm LC cells with planar alignment layers and ITO coating using capillary forces.

### 2.2. Design and Fabrication

**Figure 1:**
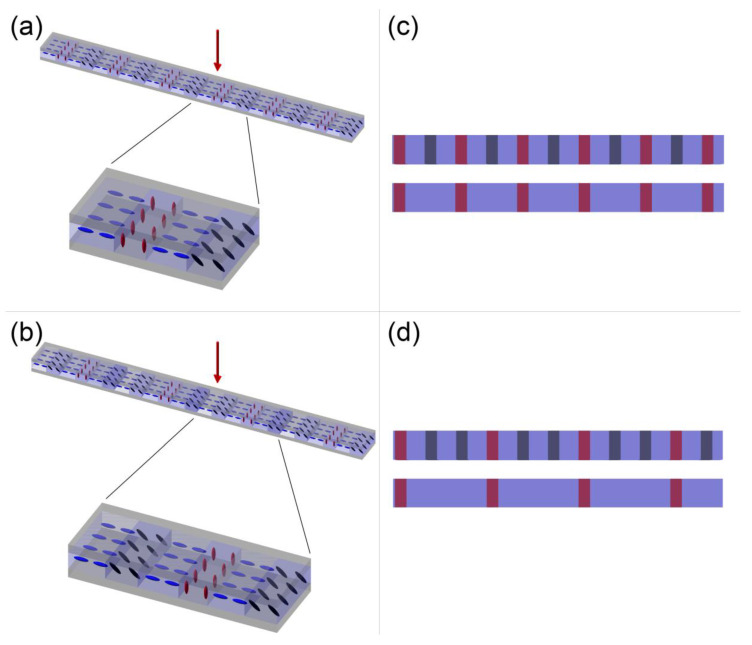
Schematic representation of the designed diffraction gratings. The schemes (**a**,**b**) demonstrate the molecular arrangement in each type of diffraction grating, as well as zoom on each grating’s building block consisting of: (**a**) 4 elements (double period change), (**b**) 6 elements (triple period change). The red arrows represent the direction of laser light incident on diffraction gratings during examination in optical setup. The schemes (**c**,**d**) visualize the polarization-dependent period change in grating (**a**) and (**b**), respectively.

The samples were fabricated by selective irradiation (λ = 405 nm, I = 1 mW/cm^2^, 15 min per slit type) with periodic patterns corresponding to the grating’s slits with each designed molecular arrangement of the LC. During polymerization, the LC cell was connected to a function generator to control the orientation of LC molecules. The building block of each diffraction grating consisted of 4 or 6 elements of identical width corresponding to the grating’s slits, which is demonstrated in Figure 1. Regardless of structure design, every other slit was left non-polymerized (planar) and the LC molecules in the other ones were polymerized either under 20 V to obtain a homeotropic orientation or under lower voltage to achieve only a tilt of the molecules with respect to the LC cell plane. The LC arrangement in case of a 4-element building block (Figure 1a) was as follows: planar, polymerized with a tilt, planar, polymerized in homeotropic orientation. In case of a 6-element block (Figure 1b), there were two additional slits: planar and polymerized with a tilt. Periodically varying orientations of LC molecules resulted in periodic changes in effective birefringence between the grating’s slits. Consequently, linearly polarized light passing through each type of slit experienced a different phase delay. Moreover, the effective birefringence, and thus the introduced phase delay, depends on the relation between the polarization direction and LC director in each slit. The proposed design enabled the creation of gratings with either double (Figure 1c) or triple (Figure 1d) period changes with polarization rotation by ensuring that the phase difference between planar slits and the ones with a tilt of LC molecules was equal to integer multiplicity of π for a specific polarization direction.

### 2.3. Analysis of Diffraction Patterns

The performance of diffraction gratings was analyzed in a free space optical setup consisting of a laser emitting a beam with 660 nm center wavelength as a light source and a half-wave plate to control the polarization direction of light incident on the sample. The diffraction patterns were observed on a screen set in a distance from the sample that was sufficient to fulfil the small angle approximation (sinα≅tgα). The measurements of grating’s period values were conducted under a digital microscope (Keyence VHX 5000, Tokyo, Japan) with a sample positioned between crossed polarizers. To ensure that the measured period values corresponded to the observed diffraction patterns, the period values were also calculated based on the measured distances between the 0th and 1st diffraction order by approximating the formula:

dsinα=mλ,

where d—grating period, α—diffraction angle, m—diffraction order, and λ—illuminating wavelength. The distances between higher diffraction orders were not considered, as for larger diffraction angles, the sinα≅tgα approximation would not be valid.

## 3. Results and Discussion

### 3.1. Diffraction Gratings with Double Period Change

**Figure 2:**
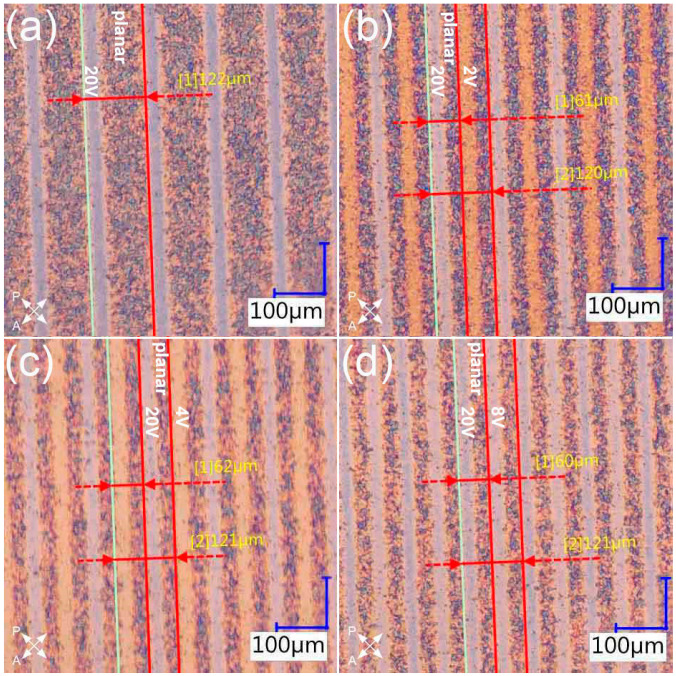
Comparison of fabricated diffraction gratings observed under a polarizing microscope with crossed polarizers. The reference grating (**a**) has every other slit polymerized under 20 V. The gratings (**b**–**d**) have half of the polymerized slits irradiated under 20 V and the rest is polymerized under: (**b**) 2 V, (**c**) 4 V, and (**d**) 8 V. The remaining slits are not polymerized. Period(s) of each grating are measured with the measurement tools of the microscope.

The first samples were designed to exhibit doubling of the grating’s period. The polymerized slits were irradiated interchangeably in two different orientations of LC molecules (homeotropic and tilted); however, the voltage values corresponding to the tilt angle were chosen arbitrarily. This step was aimed at determining the voltage that provided a sufficient tilt of LC molecules to allow for a period change for a specific polarization direction. The fabricated gratings, including the reference one with only larger period, are presented in Figure 2.

**Figure 3:**
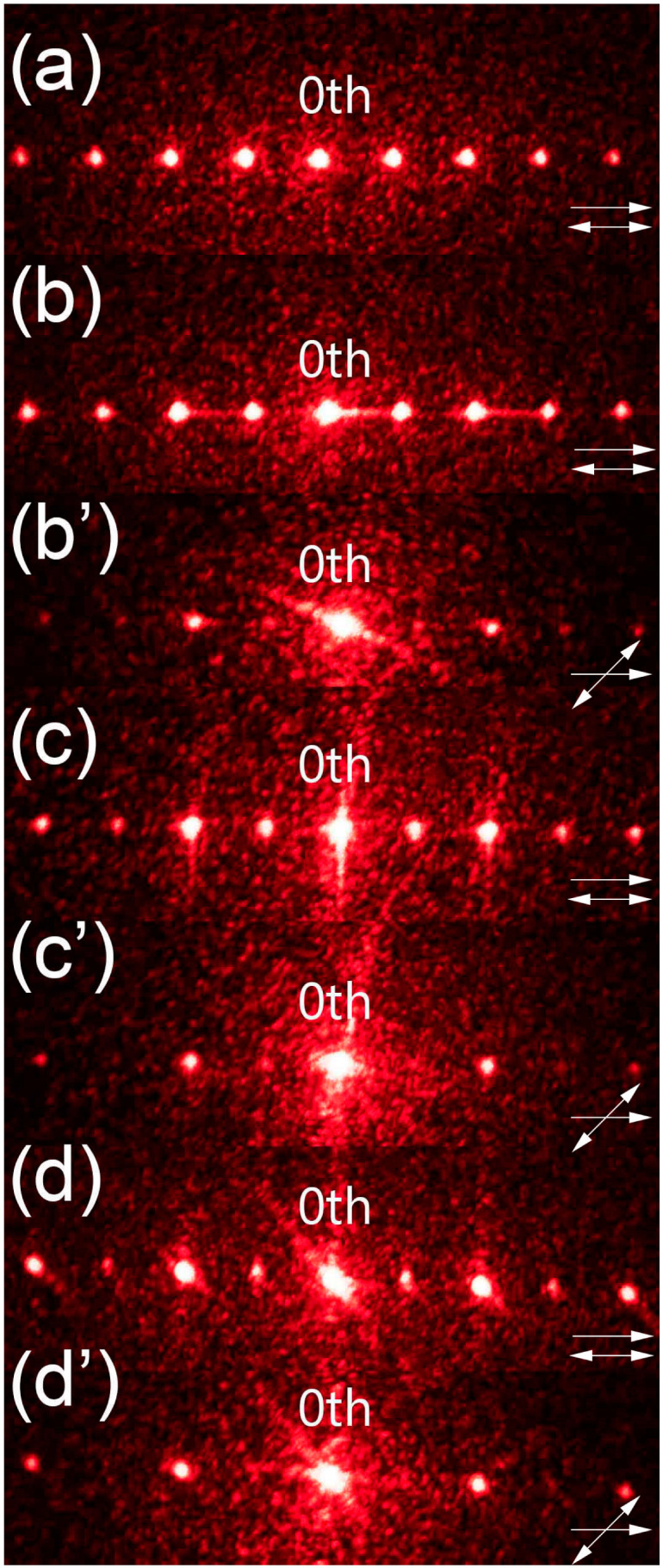
Comparison of diffraction patterns observed for the gratings presented in Figure 2. The reference grating (Figure 2a) has only one period, so the diffraction pattern is presented for only one polarization direction (picture (**a**)). The other gratings are examined for different polarization directions to observe the period change: (**b**,**b’**) Figure 2b, (**c**,**c’**) Figure 2c, and (**d**,**d’**) Figure 2d. The relation between LC director in planar slits and polarization direction is marked in each image. For all the images, 80 pixels correspond to 1 cm on the screen.

The obtained samples were examined in a free space optical setup and the observed diffraction patterns were compared with the one acquired for the reference sample (Figure 3).

The diffraction patterns obtained for the dual-period samples (Figure 3c–d’) clearly demonstrate that it was possible to find a linear polarization direction for which the incident light experienced both a smaller period and the larger one that was identical to the reference sample. In case of the reference diffraction pattern (Figure 3a), the diffraction efficiency was gradually decreasing for higher diffraction orders. However, for all the other samples (Figure 3b,c,d), the diffraction efficiency for even diffraction orders was higher than for the odd ones. This disproportion in diffraction efficiency was larger for the samples with larger tilt of LC molecules (higher voltage values during polymerization), and such behavior was due to phase mismatch between the non-polymerized slits and half of the polymerized ones. When it comes to the smaller period, it was possible to observe diffraction patterns corresponding to it only for two samples (Figure 3c’,d’). In the case of the sample with part of the slits polymerized under 2 V (Figure 2b), it was not possible to find a polarization direction for which the change in period could have been observed—the diffraction pattern corresponding to the larger period was visible regardless of polarization direction and the change was observed only in diffraction efficiency (Figure 3b,b’). It was thought that the tilt of LC molecules in the regions polymerized under 2 V was too small, and thus a necessary phase difference between those slits and the planar ones could not have been obtained. The highest diffraction efficiency in the case of the smaller period was obtained for the sample with part of the slits polymerized under 8 V (Figure 3d’), which was most likely due to the largest phase difference between those slits and the planar ones. However, improvement in the diffraction efficiency for the smaller period resulted in worsening of the grating’s performance in the case of a larger period. Moreover, the phase delays between all the slit types could not have been equalized for any polarization direction whereas, in theory, it should be possible for polarization direction perpendicular to LC director in the planar slits. This was due to changes in LC’s refractive index resulting from the formation of polymer network [7]. As a result, the diffraction patterns corresponding to smaller grating period were observed for polarization direction at 45°+m·90° with respect to LC director in the planar slits. The patterns corresponding to the larger one were visible for most angles; however, the best results were achieved for m·90° angles between LC director in the planar slits and polarization direction. Overall, the optimal results were obtained for the grating presented in Figure 2d as both a clear period change and high diffraction efficiency for smaller period were achieved (Figure 3d,d’).

To ensure that the observed diffraction patterns corresponded to the measured period values of the fabricated diffraction gratings, the period values calculated based on the measured distance between the 0th and 1st diffraction order were compared with the measured ones for each sample. The measurement results, as well as the comparison of period values, can be found in Table 1.

The results presented in Table 1 prove that the observed diffraction patterns corresponded to the measured period value(s) of each grating within the measurement uncertainty. The differences between the measured and calculated values were within the measurement accuracy. Moreover, small variations (1–2 µm) in measured period values between the samples were due to microscope’s measurement accuracy and slight imperfections on the edges of polymerized patterns.

### 3.2. Diffraction Gratings with Triple Period Change

**Figure 4:**
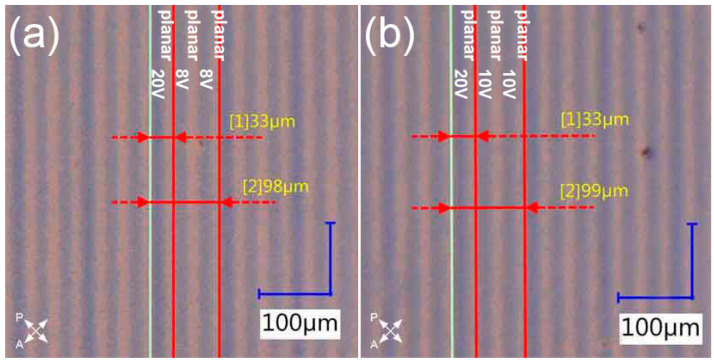
Comparison of fabricated diffraction gratings observed under a polarizing microscope with crossed polarizers. In both cases, every third polymerized slit is polymerized under 20 V, and the other ones are polymerized under: (**a**) 8 V and (**b**) 10 V. The remaining slits are not polymerized. Periods of each grating are measured with the measurement tools of the microscope.

The optimal results obtained previously were the basis for design of the gratings with triple period change. The only difference, compared to the previous design, was that in this case tripling of the period for a specific polarization direction of incident light was desired, and thus every third polymerized slit was irradiated under 20 V. The voltage values for other slits were chosen based on the previous experiments—for one sample, it was 8 V, as this value gave optimal results, and for the other one, it was 10 V, to check how the grating’s performance would change. It was observed that the fabricated samples looked nearly identical under a polarizing microscope, as shown in Figure 4.

**Figure 5:**
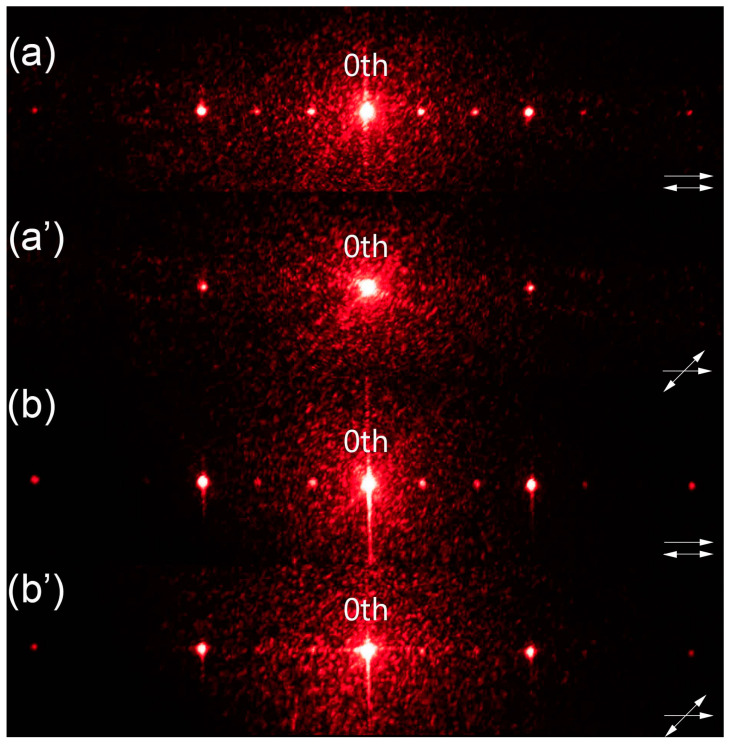
Comparison of diffraction patterns observed for the gratings presented in Figure 4. The gratings are examined for different polarization directions to observe the period change: (**a**,**a’**) Figure 4a and (**b**,**b’**) Figure 4b. The relation between LC director in planar slits and polarization direction is marked in each image. For all the images, 80 pixels correspond to 1 cm on the screen.

As was performed previously, the gratings were examined in a free space optical setup to investigate their performance and determine if there were any differences between them. The behavior of the sample polymerized under 8 V was consistent with previous results. It was possible to find a polarization direction for which the incident light behaved like the slits with a tilt of LC molecules were not present (Figure 5a), and the one for which the diffraction pattern corresponded to the smaller period (Figure 5a’). The performance of the second sample was very similar; however, small differences were observed. The diffraction efficiency for the smaller period was higher compared to Figure 5a’, to the point where it was possible to observe the 2nd diffraction order (Figure 5b’). On the other hand, there were visible traces of the 1st diffraction order corresponding to the larger period in the diffraction pattern observed for the smaller period. This result indicated that voltage values equal to 8 V or slightly higher than this would be suitable for obtaining optimal grating’s performance. Moreover, the disproportion in diffraction efficiencies between diffraction orders in the case of larger period was observed for both samples (Figure 5a,b). This type of behavior was consistent with previous results. The only difference was that in this case every third diffraction order had higher efficiency, which was an expected outcome.

Additionally, the consistency between the observed diffraction patterns and the measured period values was examined as previously. The results demonstrated in Table 2 prove that the measurements agreed with the calculations within the uncertainty as predicted.

## 4. Conclusions

The results presented in this paper show that the polarization-dependent properties of LC-based diffractive optical elements can be utilized to create dual-period diffraction gratings operating for linearly polarized light. The gratings were created by selective photopolymerization of the slits with different orientations of LC molecules. This allowed us to observe diffraction patterns corresponding either to smaller or larger grating’s period by changing of azimuth of linear polarization of incoming light. It was possible to fabricate diffraction gratings with either double or triple change of the period when voltage value corresponding to the tilt of LC molecules in part of the slits was chosen appropriately. Moreover, it was observed that improvement in the diffraction efficiency for a smaller period resulted in higher disproportion in diffraction efficiency between diffraction orders in case of a larger period. The presented method could be adopted to fabricate dual-period diffraction gratings with higher multiplier of the base period (i.e., four, five, or more times larger than base period). Smaller base periods could be obtained by using higher precision of selective photopolymerization, i.e., high resolution direct laser writing. One of our long-term goals is to optimize the composition of LC material to reduce the scattering of light on the polymer network and utilize such dual-period gratings for fabrication of a compact spectrometer with switchable resolution and measurement range, where the user can change the range/resolution just by simple mechanical switch connected with properly oriented polarizers. 

## Figures and Tables

**Table 1:** Comparison of the calculated and measured period(s) of the gratings presented in Figure 2 for two polarization directions corresponding to different period values. The distance between the sample and the screen was 270.00 (0.58) cm.

Grating	Period Measured under a Microscope [µm]	Measured Distance between 0th and 1st Diffraction Order [cm]	Period Calculated Based on Diffraction Pattern [µm]
20 V	122.0 (1.2)	1.463 (0.014)	121.9 (2.8)
2 V/20 V	61.0 (1.2)	2.938 (0.014)	60.7 (1.4)
	120.0 (1.2)	1.463 (0.014)	121.8 (2.8)
4 V/20 V	62.0 (1.2)	2.938 (0.014)	60.7 (1.4)
	121.0 (1.2)	1.463 (0.014)	121.8 (2.8)
8 V/20 V	60.0 (1.2)	2.950 (0.014)	60.4 (1.4)
	121.0 (1.2)	1.463 (0.014)	121.8 (2.8)

**Table 2:** Comparison of the calculated and measured periods of the gratings presented in Figure 4 for two polarization directions corresponding to different period values. The distance between the sample and the screen was 270.00 (0.58) cm.

Grating	Period Measured under a Microscope [µm]	Measured Distance between 0th and 1st Diffraction Order [cm]	Period Calculated Based on Diffraction Pattern [µm]
8 V/20 V	33.0 (1.2)	5.363 (0.014)	33.23 (0.75)
	98.0 (1.2)	1.800 (0.014)	99.0 (2.3)
10 V/20 V	33.0 (1.2)	5.400 (0.014)	33.00 (0.75)
	99.0 (1.2)	1.800 (0.014)	99.0 (2.3)

## Data Availability

Data are contained within the article.
